# HDAC I inhibition in the dorsal and ventral hippocampus differentially modulates predator-odor fear learning and generalization

**DOI:** 10.3389/fnins.2015.00319

**Published:** 2015-09-22

**Authors:** Robin K. Yuan, Jenna C. Hebert, Arthur S. Thomas, Ellen G. Wann, Isabel A. Muzzio

**Affiliations:** ^1^Department of Psychology, University of PennsylvaniaPhiladelphia, PA, USA; ^2^Biological Basis of Behavior, University of PennsylvaniaPhiladelphia, PA, USA; ^3^Department of Biology, University of PennsylvaniaPhiladelphia, PA, USA; ^4^Department of Biology, University of Texas at San AntonioSan Antonio, TX, USA

**Keywords:** histone deacetylase inhibitors, MS-275, contextual fear conditioning, fear generalization, hippocampus, predator odor

## Abstract

Although predator odors are ethologically relevant stimuli for rodents, the molecular pathways and contribution of some brain regions involved in predator odor conditioning remain elusive. Inhibition of histone deacetylases (HDACs) in the dorsal hippocampus has been shown to enhance shock-induced contextual fear learning, but it is unknown if HDACs have differential effects along the dorso-ventral hippocampal axis during predator odor fear learning. We injected MS-275, a class I HDAC inhibitor, bilaterally in the dorsal or ventral hippocampus of mice and found that it had no effects on innate anxiety in either region. We then assessed the effects of MS-275 at different stages of fear learning along the longitudinal hippocampal axis. Animals were injected with MS-275 or vehicle after context pre-exposure (pre-conditioning injections), when a representation of the context is first formed, or after exposure to coyote urine (post-conditioning injections), when the context becomes associated with predator odor. When MS-275 was administered after context pre-exposure, dorsally injected animals showed enhanced fear in the training context but were able to discriminate it from a neutral environment. Conversely, ventrally injected animals did not display enhanced learning in the training context but generalized the fear response to a neutral context. However, when MS-275 was administered after conditioning, there were no differences between the MS-275 and vehicle control groups in either the dorsal or ventral hippocampus. Surprisingly, all groups displayed generalization to a neutral context, suggesting that predator odor exposure followed by a mild stressor such as restraint leads to fear generalization. These results may elucidate distinct functions of the dorsal and ventral hippocampus in predator odor-induced fear conditioning as well as some of the molecular mechanisms underlying fear generalization.

## Introduction

Predator odors are ethologically relevant stimuli that have been shown to elicit a variety of defensive responses in rodents (Blanchard and Blanchard, 1990; Zangrossi and File, 1992; Wallace and Rosen, 2000; Dielenberg and McGregor, 2001; Wang et al., 2013a), and, under some conditions, can also produce conditioning (Blanchard et al., 2001; Dielenberg et al., 2001; Takahashi et al., 2008). Contextual fear conditioning, including predator odor fear learning, involves the association of a context (the conditioned stimulus, CS) with a predator odor (unconditioned stimulus, US), which leads to the emergence of a conditioned fear response (CR) in response to the context CS (Fanselow, 2000; Maren and Holt, 2000; Anagnostaras et al., 2001; Rosen, 2004). We have recently developed and characterized a predator odor fear conditioning paradigm using coyote urine that is effective with mice. We showed that this paradigm produces moderate but consistent freezing, a stereotypic response to fear observed in rodents, during long-term retrieval tests. This response is not observed when animals are exposed to water (no odor) or an aversive non-fearful odor (2-methyl butyric acid), indicating that the freezing is a result of associative learning (Wang et al., 2013a, 2015). Furthermore, the conditioned fear response is context specific since freezing is observed only in the training context, and it requires both the dorsal and ventral hippocampus (Wang et al., 2013a).

Using this paradigm, we recently found that spatial representations formed in the dorsal hippocampus after predator odor fear conditioning are stable in the long term (Wang et al., 2012) but become unstable again during extinction (Wang et al., 2015), suggesting that predator odor learning alters the stability of the dorsal hippocampal representation of context. These findings correlate with numerous studies indicating that the dorsal hippocampus receives preprocessed spatial information (for review, see Witter et al., 2014) and thus plays a critical role forming representations of context during conditioning (for review, see Maren and Holt, 2000); however, the role of the ventral region remains unclear. Clarifying the role of the ventral hippocampus for predator odor fear learning is particularly important because this region receives most of the olfactory inputs from the medial and posterior amygdala (Pitkanen et al., 2000; Kemppainen et al., 2002), areas that receive projections from the main and accessory olfactory system involved in predator odor processing (Masini et al., 2010). Moreover, in addition to these neuroanatomical differences, ventral and dorsal cells display distinct firing characteristics, further suggesting that these regions may have different functions. Cells in the dorsal hippocampus fire in specific circumscribed locations, whereas ventral cells have large and overlapping receptive fields (O'Keefe and Dostrovsky, 1971; Kjelstrup et al., 2008; Keinath et al., 2014). Based on these differences, it has been suggested that the dorsal region may be important for minimizing memory interference by coding specific aspects of contexts, while the ventral hippocampus may play a role in contextual generalization (Komorowski et al., 2013; Keinath et al., 2014). However, no studies have directly tested if these differential functions play a role in contextual fear learning.

On the molecular level, the formation of new memories requires alterations in gene transcription, which lead to the translation of proteins necessary for the cellular changes implicated in long-term memory (for review, see Kandel, 2012). This occurs through modifications of chromatin, a DNA-protein complex. The basic unit of chromatin is the nucleosome, which consists of DNA wrapped around four histone proteins. Modifying these proteins through processes such as acetylation, phosphorylation, and methylation changes the state of the chromatin, influencing the rate of transcription by making the DNA more or less accessible to transcription factors (Levenson and Sweatt, 2005; Wood et al., 2006). The most studied and well understood of these modifications in relation to memory is histone acetylation, a process that facilitates gene transcription by relaxing chromatin structure. This, in turn, leads to synthesis of proteins necessary for long-term memory (Peixoto and Abel, 2013). Histone acetylation is regulated by histone acetyltransferases (HATs) and histone deacetylases (HDACs), enzymes that increase and decrease acetylation, respectively (for review, see Levenson and Sweatt, 2005; Day and Sweatt, 2011). Evidence from experiments investigating the correlation between histone modifications and long-term memory in mice suggests that changes in acetylation are essential for hippocampus-dependent fear learning using electrical shock (Vecsey et al., 2007; Bahari-Javan et al., 2012). However, it is unknown what role histone acetylation plays in predator odor conditioning and whether the dorsal and ventral hippocampus differentially respond to chromatin alterations.

Here, we investigated the effects of the HDAC inhibitor MS-275, a class I-specific inhibitor (Simonini et al., 2006; Beckers et al., 2007; Khan et al., 2008; Formisano et al., 2015), in the dorsal and ventral hippocampus on innate anxiety and during predator odor fear conditioning. We found that MS-275 had no effect on traditional anxiety tests. However, injections of MS-275 after context pre-exposure (pre-conditioning injections) had different roles in the dorsal and ventral hippocampus, leading to enhanced fear and generalization, respectively. Interestingly, although injections after conditioning did not have effects in any of the groups, all conditions displayed fear generalization, suggesting that animals generalize fear to neutral contexts when a stressor, such as restraint, follows immediately after predator odor exposure. These results extend our understanding of hippocampal function during fear learning and provide insights about the learning contingencies that could lead to fear generalization.

## Materials and methods

### Animals

Male C57BL/6 mice 2–5 months of age (Jackson Laboratories, Bar Harbor, ME) were housed individually, kept on a 12-h light/dark cycle, and allowed access to food and water *ad libitum* for at least 1 week prior to beginning behavioral experiments. All experiments were approved by the Institution of Animal Care and Use Committee of the University of Pennsylvania, and were carried out in accordance with NIH guidelines.

### Anxiety measures

Animals were run in the open-field test and black-white box to determine the effects of MS-275 on innate anxiety. MS-275 was infused bilaterally into either the dorsal or ventral hippocampus 1–2 h before conducting anxiety tests. For the open field test, mice were placed in the center of a large cylindrical arena (70 cm in diameter). The arena was illuminated by a ceiling-mounted array of eight 60-watt lights arranged symmetrically around the perimeter, approximately 1.8 m above the base. Explorative behavior was recorded for 20 min. Additionally, we evaluated freezing by calculating the percent time the animals remain immobile, except for respiratory movements. For the black/white two compartment box, we used a plastic box divided into two equal compartments (22 × 24 cm) connected by a small opening. The black compartment was darkened with black contact paper and covered with a piece of cardboard, while the open-topped white compartment was lined with white contact paper and illuminated by three ceiling-mounted 60-watt lights approximately 1 m above the apparatus, aimed at the center of the compartment. Mice were placed in the center of the white compartment facing the black side, and explorative behavior was recorded for 3 min (see behavioral analysis for quantification details below).

### Contextual fear conditioning and context discrimination

Prior to the start of behavioral experiments, animals were handled and restrained twice a day for 2 consecutive days. Animals were then conditioned using a predator odor contextual fear conditioning paradigm previously characterized in our lab (Wang et al., 2013a). We have demonstrated in several studies that this paradigm produces moderate but consistent increases in freezing, which are not seen when animals undergo the same schedule of context exposures with no odor exposure or with exposure to a non-fearful odor (Wang et al., 2012, 2013a, 2015). On day 1 (one day before conditioning), mice were habituated to a cylindrical training context (baseline context A, blA) and an equivalently sized neutral context (baseline context B, blB) for 10 min each. Both contexts were 35 cm in diameter and had distinct configurations of black visual cues on the cylinder's white walls; additionally, the contexts were placed in separate rooms. The next day (day 2, 24 h after baseline context exposures), a paper towel square (2 × 2 cm) saturated with 40 drops of coyote urine (Maine Outdoor Solutions, Harmon, MN) was placed in the center of context A, and mice were re-exposed to the context for 4 min in the presence of the odor (conditioning session, cond). A short-term retrieval test was conducted 1 h later in context A without odor for 10 min. We have previously shown that this retrieval session is important for the consolidation of the fear representation in the long term (Wang et al., 2012). The following day (day 3, 24 h after conditioning), a long-term retrieval test was conducted in both context A and context B for 10 min without odor (24h A and 24h B). The order of context A and context B during baseline and the 24 h retrieval test was counterbalanced across animals. At all time points other than the conditioning session, a paper towel square saturated with water was placed in the center of the context. MS-275 or DMSO (4%) was administered bilaterally into either the dorsal or ventral hippocampus immediately following the baseline context exposures (pre-conditioning injections) or immediately after the conditioning session (cond; post-conditioning injections). We evaluated freezing as a measure of learning as described below.

### Behavioral analysis

All behavioral measures were recorded and analyzed using the Limelight automated tracking system (Coulburn Instruments). For the open-field test, the context was divided into three equally spaced concentric circles and the percent time spent in these areas was measured. For the black-white box, the percent time spent in the white compartment of the white/black box was measured, along with the number of reentries to the white side. For conditioning and the open field, freezing was quantified as the percentage of time during which the velocity of the animal was lower than 0.6 cm/s. Freezing was evaluated using both Lime Light and custom-written MATLAB code. For the MATLAB analysis, the position data were smoothed with a 1 s boxcar to eliminate jitter in the tracking. Finally, we calculated percent freezing by calculating the percent ratio between freezing at 24 h and freezing during baseline for each context.

### Surgery

Mice were anesthetized with a mixture of ketamine (100 mg/kg) and xylazine (10 mg/kg) administered intraperitoneally and placed on a stereotaxic apparatus in a flat skull position (David Kopf Instruments, Tujunga, CA). 26 gauge guide cannulas (Plastics One, Roanoke, VA) were implanted bilaterally in either the dorsal or ventral hippocampus at the following coordinates, measured from Bregma in mm. Dorsal: AP, -1.7; ML, ± 1.5; DV: -1.0 (internal cannulas project an additional 0.7 mm beyond guides for an injection depth of -1.7). Ventral: AP, -3.0; ML, ± 2.8; DV: -2.0 (internal cannulas project an additional 1.5 mm beyond guides for an injection depth of -3.5). An anchor screw was placed just anterior to lambda, and cannulas were affixed to the skull with cyanoacrylate and dental cement. After surgery, animals were allowed to recover for 1 week prior to behavioral experiments.

### Bilateral hippocampal injections and drug concentration in the hippocampus

MS-275 (SelleckChem, Houston, TX) was diluted to 1 mM using 4% dimethyl sulfoxide (DMSO) in ACSF, then infused bilaterally into either the dorsal or ventral hippocampus through the implanted guide cannulas using a standard infusion syringe pump (Harvard Apparatus, Holliston, MA). Total volume injected was 0.5 μl on each side at a rate of 0.5 μl/min. Controls were injected with an equivalent volume of vehicle (4% DMSO in artificial cerebrospinal fluid). We estimated the concentration of MS-275 in the hippocampus to be roughly 71 μM, since the average volume of the hippocampus in C57bl6 mice is 28 mm^3^, with each hemisphere being approximately 14 mm^3^ and each dorsal and ventral sub-regions around 7 mm^3^ (Peirce et al., 2003). This concentration is well above the dosages that are effective in inhibiting class I HDACs (Hu et al., 2003; Khan et al., 2008). The increase in acetylation produced by MS-275 (Simonini et al., 2006; Formisano et al., 2015) is evident 2 h after drug treatment and persists for up to 8 h (Simonini et al., 2006). Injections were performed in a room separate from all behavioral experiments and were given after contextual pre-exposure (pre-conditioning injections) or after predator odor exposure (post-conditioning injections).

### Histology

To verify cannula placements, animals were sacrificed after behavioral experiments. Brains were removed and fixed at 4°C with 10% formalin for at least 24 h. They were then transferred to a 30% sucrose solution and kept for at least 48 h at 4°C for cryoprotection. Brains were then cryosectioned (35 μm, coronal) and Nissl stained with cresyl violet using standard histological procedures (Powers and Clark, 1955).

### Statistics

Independent *t*-tests were used to evaluate anxiety measures. Two-Way ANOVAS with repeated measures were used to compare baseline and post-conditioning freezing in the training and neutral context. Student Newman Keuls *post hoc* tests were used to determine which groups were significantly different. Independent and paired *t*-tests were used to evaluate percent of freezing relative to baseline.

## Results

### MS-275 has no effect on innate anxiety

**Figure 1:**
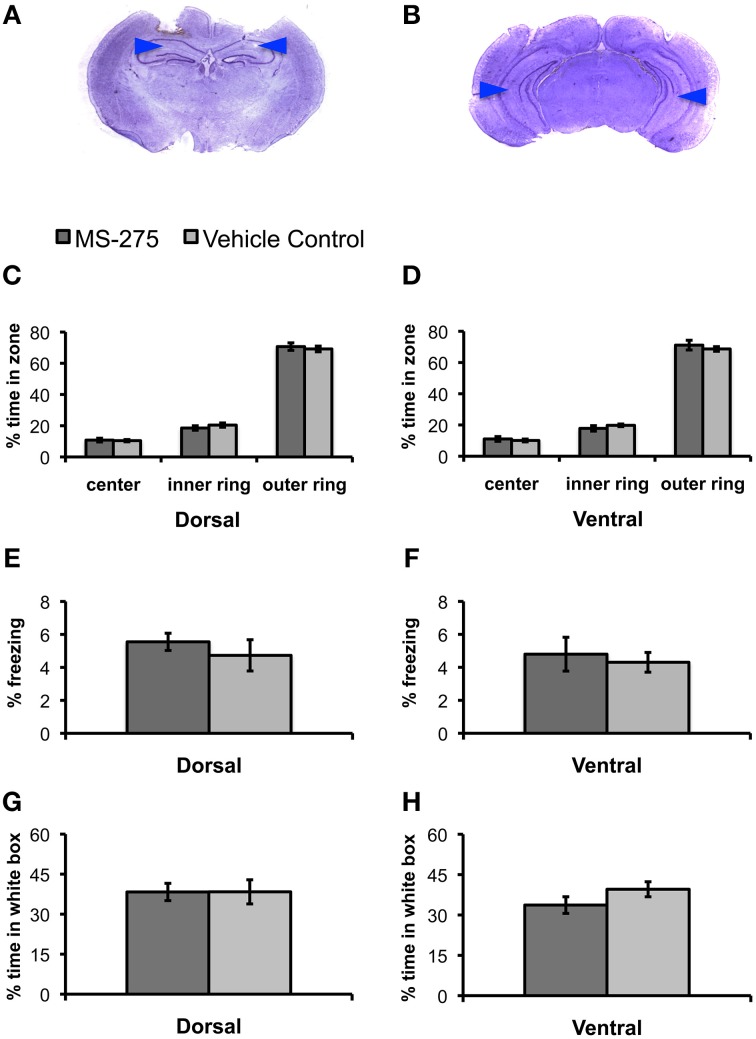
**(A,B)** Photomicrograph of Nissl-stained coronal section showing position of dorsal **(A)** and ventral **(B)** guide cannulas. Note that internal cannulas for dorsal injections project an additional 0.7 mm beyond guide cannulas, and internal cannulas for ventral injections project an additional 1.5 mm beyond guide cannulas. Blue arrows indicate estimated injection depth. **(C–H)** Animals injected with MS-275 or vehicle in either the dorsal or ventral hippocampus showed no significant differences in tests of anxiety. **(C–F)** Animals injected with MS-275 (dorsal: *N* = 20; ventral: *N* = 14) or vehicle (dorsal: *N* = 21; ventral: *N* = 20) showed no significant differences in behavior in the open field test. This was evident in the percentage of time spent in the center, inner ring, and outer ring of an open field in the dorsal **(C)** and ventral **(D)** groups, as well as the level of freezing observed in the open field in the dorsal **(E)** and ventral **(F)** groups. **(G,H)** Animals injected with MS-275 (dorsal: *N* = 12; ventral: *N* = 11) or vehicle (dorsal: *N* = 12; ventral: *N* = 11) in the dorsal **(G)** or ventral **(H)** hippocampus showed no significant difference in the percentage of time they spent in the white compartment of the black/white box. Means ± SEM are shown.

Since we wanted to establish the effects of HDAC inhibition on fear learning, we first investigated whether MS-275 had any effects on innate anxiety. We performed bilateral injections of MS-275 or vehicle into the dorsal or ventral hippocampus of animals 2–3 h prior to behavioral testing in the open field and the black/white box (Figures 1A,B). The open field test, which consists of free exploration in a large arena, evaluates the anxiety that rodents exhibit in open spaces. It is well established that rodents find the inner areas of the open field more anxiogenic than the outer areas (Hall, 1934; Prut and Belzung, 2003). Thus, differences in the amount of time spent in these areas normally reflect distinct levels of anxiety. Thirty-four animals were injected with MS-275 (dorsal: *N* = 20; ventral: *N* = 14), and 41 animals were injected with vehicle (dorsal: *N* = 21; ventral: *N* = 20). We divided the open field in three concentric areas and calculated the time spent in each of these zones. We did not find any differences between the groups in the percentage of time spent in the three concentric regions comprising the open field (Figures 1C,D; dorsal: center: *p* = 0.80, inner: *p* = 0.36, outer: *p* = 0.62; ventral: center: *p* = 0.57, inner: *p* = 0.30, outer: *p* = 0.46). Additionally, we did not find differences in the levels of freezing in the open field (Figures 1E,F; dorsal: *p* = 0.45; ventral: *p* = 0.66).

Next, we evaluated the effects of MS-275 on behavior in a black/white two-compartment box (Crawley and Goodwin, 1980; Sánchez, 1997). The animals are initially placed in the open white compartment, and the time they spend in this compartment vs. the dark covered side of the chamber is measured while animal freely move across these areas. Rodents find bright, open environments more anxiogenic than closed, dark ones (Sánchez, 1995); therefore, increased anxiety is typically seen through a reduction in the amount of time spent exploring the white compartment. Twenty-three animals were injected with MS-275 (dorsal: *N* = 12; ventral: *N* = 11) and 23 animals were injected with vehicle (dorsal: *N* = 12; ventral: *N* = 11). We did not find significant differences between the groups in the percentage of time spent in the white compartment (Figures 1G,H, dorsal: *p* = 0.99; ventral: *p* = 0.17) or the number of reentries to the white compartment (dorsal: *p* = 0.29; ventral: *p* = 0.24; data not shown). These results suggest that MS-275 does not affect innate anxiety in the dorsal or ventral hippocampus.

### Effects of MS-275 on fear learning

#### Inhibition of class I HDACs following contextual pre-exposure produces enhanced fear in the dorsal hippocampus and generalization in the ventral region

Several researchers have suggested that successful contextual conditioning consists of two stages. In the first stage, a representation of the context is formed, while in the second stage, the context is associated with the US (Young et al., 1994; Fanselow and Rudy, 1998; Rudy and O'Reilly, 1999, 2001; Fanselow, 2000; O'Reilly and Rudy, 2001; Rudy et al., 2004). This view is based on the observation that animals display learning deficits in the absence of contextual pre-exposure (Fanselow, 1990). Our predator odor paradigm is ideal for testing the contributions of the hippocampus to these two learning stages because animals are exposed to the context one day prior to conditioning, which provides an optimal time window to explore the effects of HDAC inhibition either during the context pre-exposure (pre-conditioning) or the associative phase of learning (post-conditioning).

**Figure 2:**
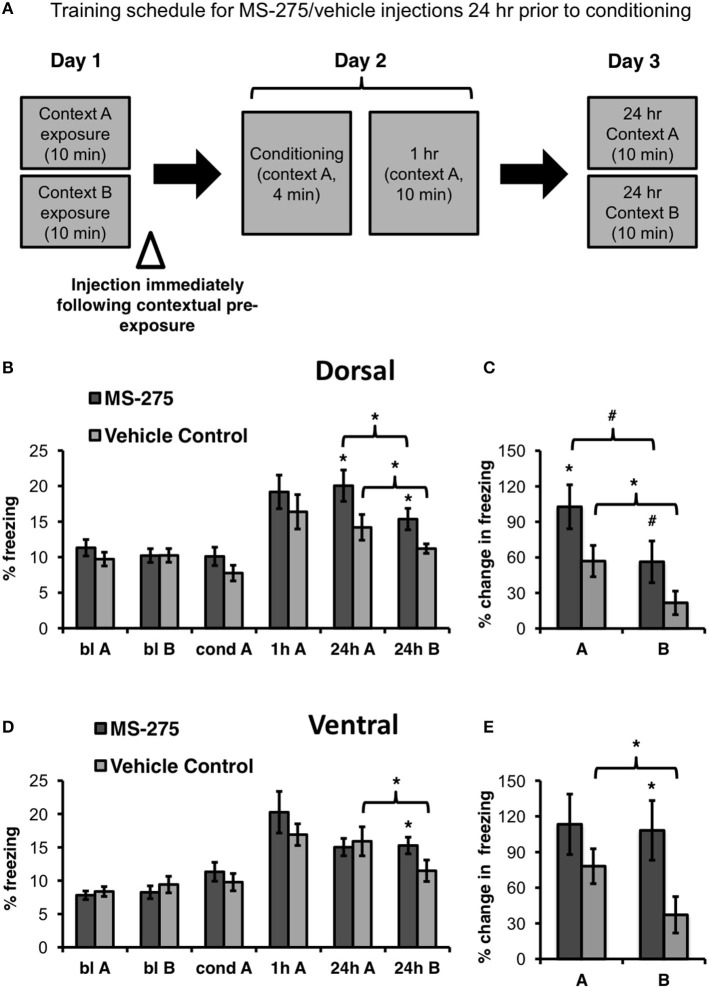
**(A)** Schematic representation of behavioral paradigm and timing of injections. Animals were injected with MS-275 or vehicle immediately following exposure to contexts A and B, 24 h prior to fear conditioning. **(B)** Animals injected with MS-275 (*N* = 13) in the dorsal hippocampus after contextual pre-exposure (bl A, bl B) exhibited enhanced fear learning in context A and elevated freezing in context B 24 h after conditioning in comparison to the vehicle-injected controls (*N* = 16). However, freezing was significantly higher in context A, the conditioning context, than in the neutral context B, indicating that these animals still discriminated between the environments. **(C)** Animals injected with MS-275 in the dorsal hippocampus exhibited a significantly greater percent change in freezing 24 h post-conditioning in context A and a trend toward enhanced freezing in context B relative to baseline freezing in each of these contexts in comparison to controls. **(D)** Animals injected with MS-275 (*N* = 17) in the ventral hippocampus after contextual pre-exposure exhibited fear generalization without showing enhanced fear learning 24 h after conditioning; This generalization effect was not observed in control animals injected with vehicle (*N* = 15). **(E)** Animals injected with MS-275 in the ventral hippocampus exhibit significantly greater percent change in freezing 24 h post-conditioning in context B relative to baseline in that context in comparison to vehicle-injected controls. No difference in percent change in freezing was observed in the training context A between the MS-275 and vehicle control animals. Means ± SEM are shown, ^*^p < 0.05, #*p* < 0.09.

To determine the role of histone acetylation during the formation of a representation of context, MS-275 or vehicle (DMSO, 4%) was injected in either the dorsal or ventral hippocampus after exposure to the training context (baseline context A) and a neutral context (baseline context B) on Day 1 (pre-conditioning injections). Twenty-four hours after the injections (Day 2), these groups were conditioned and tested 1 h after conditioning in the training context A. On day 3, the four groups were retested in the training (A) and neutral (B) context 24 h after conditioning (long-term retrieval test; see Figure 2A). The order of exposure to context A and B during baseline and the 24 h retrieval tests was counterbalanced across animals.

Since the dorsal hippocampus has been implicated in coding specific information about contexts (Nadel et al., 2013), and cells in this region are sensitive to subtle contextual changes (Colgin et al., 2008), we hypothesized that dorsal injections of MS-275 after context pre-exposure (pre-conditioning injections) should lead to enhanced learning because animals would remember the training context in more detail. These injections, however, would not substantially decrease the ability of the animals to discriminate the training and neutral contexts because the specific information about each context would be remembered. Conversely, since spatial representations in the ventral hippocampus are large and overlapping, a characteristic that may facilitate generalization (Komorowski et al., 2013; Keinath et al., 2014), we hypothesized that ventral injections after context pre-exposure (pre-conditioning injections) would lead to fear generalization to a neutral context.

There were no differences in baseline freezing prior to conditioning in the dorsal groups (MS-275: *N* = 13; vehicle control: *N* = 16). In support of our hypothesis, we found that dorsal injections significantly increased freezing in the training context (context A) in the MS-275 group in comparison to the vehicle control group. (Figure 2B; effect of group: *F*_(1, 27)_ = 4.50, *p* < 0.05, effect of session: *F*_(3, 81)_ = 17.56, *p* < 0.001; interaction: *F*_(3, 81)_ = 2.95, *p* < 0.04). *Post hoc* Student-Newman-Keuls tests (SNKTs) showed that freezing levels were comparable in the control and MS-275 groups during baseline (blA: *p* = 0.40; blB: *p* = 0.99), but were significantly different during the post-conditioning 24 h test in context A (24 h: *p* < 0.003). Furthermore, although MS-275 also produced a significant increase in freezing in the neutral context (B) (SNKTs: 24 h B: *p* < 0.03), both the vehicle and MS-275 groups, were able to discriminate the training context A from the neutral context B, which was evident in significantly higher levels of freezing in context A (vehicle control: *p* < 0.05; MS-275: *p* < 0.005). The differences between the MS-275 and vehicle groups are also evident in a significant percent increase in freezing observed 24 h after conditioning relative to baseline in context A (Figure 2C; *t*_27_ = −2.07, *p* < 0.05) and a trend in context B (*t*_28_ = −1.82, *p* = 0.08). Again, when we compared the percent increase in freezing from baseline within each group, both the MS-275 and vehicle control groups displayed higher freezing in context A than B at 24 h relative to the freezing baseline in each context (MS-275 (trend toward significance): *t*_11_ = 2.13, *p* = 0.056; vehicle control: *t*_19_ = 2.63, *p* < 0.02). These results suggest that MS-275 enhances the memory of the training context without disrupting the ability of the animals to discriminate this context from a neutral one.

In the ventral hippocampus, we did not observe differences in baseline freezing between the groups receiving injections after contextual pre-exposure (pre-conditioning; MS-275: *N* = 17; vehicle control: *N* = 15). Even though MS-275 did not significantly affect learning, the HDAC inhibitor produced fear generalization in response to the neutral context B. This was evident in higher freezing levels in the neutral context in animals injected with MS-275 than vehicle-injected controls (Figure 2D; effect of group: *F*_(1, 31)_ = 0.09, *p* = 0.77, effect of session: *F*_(3, 89)_ = 25.19, *p* < 0.001; interaction: *F*_(3, 89)_ = 2.75, *p* < 0.05). SNKTs showed no differences prior to conditioning or after conditioning in the training context (*P* > 0.05). However, the groups were significantly different in the neutral context B (24 h B: *p* < 0.04). Furthermore, while the control animals discriminated between the training and neutral context (*p* < 0.003), the MS-275 group did not (*p* = 0.865). These effects are also evident when we examined the percent increase in freezing at 24 h after conditioning relative to baseline in each context, showing that there were no differences in the training context A (*t*_35_ = −0.97, *p* = 0.34) but significantly higher freezing in the MS-275 group in context B (*t*_35_ = 2.36, *p* < 0.03; Figure 2E). Importantly, while vehicle control animals clearly discriminated between the contexts (*t*_16_ = 2.36, *p* = 0.03), MS-275 animals showed no difference in freezing in context A and B (*t*_19_ = 0.08, *p* = 0.94). These results suggest that HDAC I inhibition in the dorsal and ventral hippocampus plays different roles in predator odor fear memory. MS-275 leads to enhanced fear memory in the dorsal hippocampus and fear generalization in the ventral region.

#### Inhibition of class I HDACs following conditioning has no effect on fear learning

**Figure 3:**
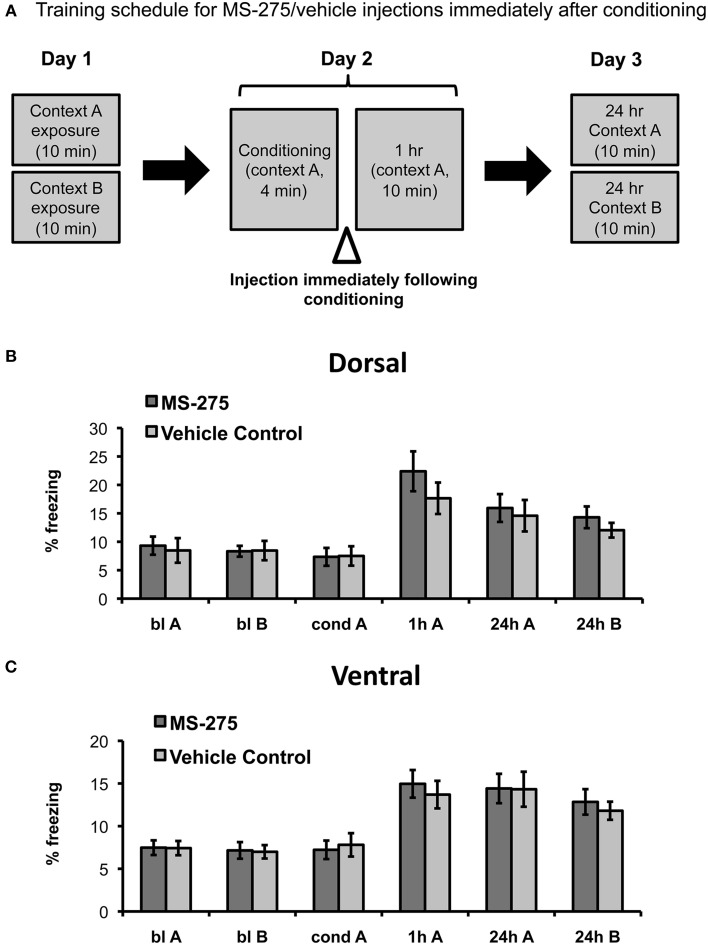
**(A)** Schematic representation of behavioral paradigm and timing of injections. Animals were injected with MS-275 or vehicle immediately following fear conditioning in context A. Animals injected in the dorsal (**B**; MS-275: *N* = 11, vehicle: *N* = 11) or ventral (**C**; MS-275: *N* = 13, vehicle: *N* = 14) hippocampus after the conditioning session exhibited fear generalization 24 h after conditioning. Means ± SEM are shown, *p < 0.05.

To determine the role of histone acetylation along the longitudinal hippocampal axis after coyote exposure, we then examined groups of mice injected with MS-275 or vehicle (DMSO, 4%) after conditioning (see Figure 3A). The amygdala has been identified as a critical brain region for the associative phase of predator odor fear learning (for review, see Takahashi et al., 2008). In particular, it has been demonstrated that the medial amygdala plays a role during acquisition (Blanchard et al., 2005; Takahashi et al., 2007) and the basolateral amygdala during consolidation (Takahashi et al., 2007). Since the dorsal hippocampus receives very few projections from the basolateral amygdala and no projections from the medial amygdala, the area where olfactory information converges (Pikkarainen et al., 1999), we hypothesized that dorsal injections of MS-275 would not affect predator odor fear learning. Conversely, the ventral hippocampus receives strong projections from the posterior, medial, and basolateral amygdala (Pitkanen et al., 2000; Kemppainen et al., 2002) as well as structures associated with the hypothalamic-pituitary adrenal axis (Witter, 1986), which suggests that the ventral hippocampus may also be involved in processing anxiety and fear. However, since we did not observe any effects of MS-275 on anxiety measures in the ventral region, we hypothesized that HDAC I inhibition after conditioning would not have an effect on the conditioning phase in the ventral hippocampus.

In agreement with our predictions, we found no significant effect of MS-275 in the dorsal hippocampus (effect of group: *F*_(1, 33)_ = 0.25, *p* = 0.62, interaction: *F*_(3, 91)_ = 0.06, *p* = 0.98). The MS-275 (*N* = 11) and vehicle control (*N* = 11) groups only showed an effect of session reflecting that freezing was significantly higher after learning (Figure 3B; effect of session: *F*_(3, 91)_ = 17.04, *p* < 0.001; *post hoc* comparisons showed that freezing was significantly higher after conditioning in comparison to baseline in both context A and B, *p* < 0.05). In the ventral groups, we observed a similar pattern. There were also no significant differences between the MS-275 (*N* = 13) and control (*N* = 14) groups (effect of group: *F*_(1, 28)_ = 0.51, *p* = 0.48, interaction: *F*_(3, 75)_ = 1.13, *p* = 0.34), though the groups displayed a significant effect of session, which reflected that freezing changed after learning (Figure 3C; effect of session: *F*_(3, 75)_ = 27.19, *p* < 0.001, *post hoc* comparisons showed that freezing was significantly higher after conditioning in comparison to baseline in both context A and B, *p* < 0.05). The fact that all groups exhibited significantly higher freezing in the neutral context B at 24 h compared to baseline indicates that generalization is not produced by HDAC inhibition. Since all animals are restrained during the injections, we suggest that the observed generalization occurs as a result of having an additional stressor following predator odor exposure. These findings indicate that predator odor fear learning provides a good model to study fear generalization.

## Discussion

Using predator odor conditioning, we tested the effects of the class I HDAC inhibitor MS-275 in the dorsal and ventral hippocampus on innate anxiety and at different stages of fear learning. We found that HDAC I inhibition after contextual pre-exposure (pre-conditioning) has different effects in the dorsal and ventral hippocampus. In the dorsal hippocampus, HDAC inhibition enhances fear learning, whereas in the ventral hippocampus, it leads to fear generalization. However, this epigenetic mechanism does not affect learning when HDAC inhibition takes place after conditioning. Instead, the presentation of a predator odor followed by a stressor results in fear generalization to a neutral context.

There are several advantages associated with the use of predator odors in fear learning. First, odors are extremely relevant cues to rodents because identifying dangerous odors is critical for survival (Brennan and Keverne, 1997; Luo et al., 2003; Restrepo et al., 2004). Second, predator odors produce innate fear in many species, and thus, are ethologically relevant models (Apfelbach et al., 2005; Rosen et al., 2008; Ferrero et al., 2011). Moreover, predator stress produces long-term changes in behavior, and these changes correlate with persistent alterations in molecular fear and stress pathways (Blanchard and Blanchard, 1989; Adamec and Shallow, 1993; Adamec et al., 1998, 2001; Wiedenmayer, 2004). Here, we demonstrate that predator urine is a good model to study the molecular mechanisms underlying fear generalization.

Field studies have shown that long-lasting smells, such as predator urine, produce long-term avoidance of spatial locations in many mammalian species (Swihart et al., 1991; Rosell, 2001), suggesting that animals can recall spatial locations where these odors have been encountered for long periods of time. In this study, as well as previous ones (Wang et al., 2012, 2013a, 2015), we found that coyote urine produces consistent but moderate levels of freezing, which, at first glance, appears surprising in the context of the effects on behavior observed in field studies. However, we have also previously demonstrated that exposure to predator urine has profound and long-lasting effects on hippocampal spatial representations. Specifically, we showed that the spatial map formed after predator odor exposure stabilizes in the long term (Wang et al., 2012), and these changes can only be reversed when animals learn to perceive the context as safe after extinction (Wang et al., 2015). These findings suggest that while conditioned freezing in response to predator urine is moderate, the neurological changes associated with fear learning induced with long lasting predator smells are persistent.

Prey animals are under significant evolutionary pressure to rapidly identify and avoid novel predators, since unguarded encounters may result in death. Consequently, it has been demonstrated that after exposure to a specific predator, many species are capable of generalizing fear responses to completely novel predators that resemble the one initially encountered (Griffin et al., 2001; Ferrari et al., 2007, 2008). It follows that it would also be important for prey animals to generalize defensive fear responses from a particular dangerous context to novel but similar contexts that may also be unsafe. Fear generalization to environments that resemble one in which a threat is originally encountered may be evolutionarily advantageous, since particular types of predators are frequently found in similar habitats. Our data suggest that epigenetic mechanisms within the ventral hippocampus play a role in this process.

It is important to note that although animals injected with MS-275 in the dorsal hippocampus after contextual pre-exposure (pre-conditioning) display increased freezing in a neutral context, the level of generalization observed in these animals is minimal in comparison to ventrally injected mice. This suggests that these animals still differentiate between the training and neutral contexts. Conversely, animals injected with MS-275 in the ventral region cannot discriminate between neutral and fearful contexts. Several studies support the idea that the ventral hippocampus may play a role in fear generalization. In rats, hippocampal place cells have receptive fields of increasing size moving from the dorsal to the ventral pole (Kjelstrup et al., 2008). We recently showed that the broadly tuned nature of the cells' receptive fields favors the involvement of this region in generalization processes (Keinath et al., 2014). Furthermore, the ability of ventral cells to generalize across situations is modulated by learning (Komorowski et al., 2013). These data suggest that in rodents, the ventral region may be critical for extracting commonalities across situations. Interestingly, studies investigating anatomical differences in humans found that in healthy adults, the anterior hippocampus (ventral in rodents) contains a smaller proportion of dentate gyrus than the posterior hippocampus (dorsal in rodents) (Malykhin et al., 2010). This distinction is remarkable because the dentate gyrus is implicated in pattern separation, the process of distinguishing between similar memories (Marr, 1971; Rolls and Kesner, 2006; Bakker et al., 2008), suggesting that the ability of the hippocampus to discriminate between similar memories may decrease along the dorso-ventral longitudinal axis. In addition, fMRI studies in humans have shown that the anterior (ventral) and posterior (dorsal) hippocampi are activated in different kinds of recall tasks. Thinking of specific spatial details of an event activates the posterior (dorsal) region, while thinking about the general location of the same event activates the anterior (ventral) area (Poppenk et al., 2013). Therefore, data from both rodents and humans suggest that the dorsal and ventral hippocampus may serve different roles in encoding a representation of context. The dorsal hippocampus encodes particular features and allows animals to discriminate between similar situations, whereas the ventral hippocampus appears to facilitate generalization processes. Here we show that the consolidation of these memories involves epigenetic mechanisms.

Our data indicate that inhibition of class I HDACs does not have an effect on innate anxiety and/or the conditioning phase of predator fear learning. Since previous studies suggest that the ventral hippocampus plays a role in anxiety (Bannerman et al., 2004; Kheirbek et al., 2013), it is possible that other epigenetic mechanims modulate anxiety in this region. MS-275 preferentially inhibits HDAC1 over HDAC2/3, and has no effect on other HDACs (Khan et al., 2008; Formisano et al., 2015). Previous studies have found that HDAC1 regulates DNA repair in neurons (Wang et al., 2013b) and modulates fear extinction (Bahari-Javan et al., 2012), HDAC2 plays an important role in several forms of spatial memory (Guan et al., 2009), and HDAC3 enhances long-term contextual fear memory (McQuown et al., 2011), all of which suggest that distinct epigenetic mechanisms modulate different aspects of memory consolidation. Interestingly, a recent study demonstrated that Class II HDAC inhibitors also regulate hippocampus-dependent learning and plasticity (Kim et al., 2012). It will be important to assess if class II HDACs play a role in the ventral hippocampus and whether this epigenetic pathway affects the conditioning phase or anxiety responses. Understanding the roles played by different hippocampal regions and epigenetic markers in fear learning may shed light on the mechanisms that lead to post-traumatic stress disorder (PTSD) and other anxiety disorders stemming from deficits in contextual learning. Here, we demonstrate that predator odor fear conditioning provides a useful paradigm for understanding these processes.

## Author contributions

JCH, AST, and EGW performed experiments; RKY performed experiments, analyzed data, and wrote the manuscript; IAM designed experiments and supervised analysis and writing of the manuscript.

## Funding

This work was supported by the National Science Foundation (NSF) CAREER award to IM (grant number 1256941) and a Ruth L. Kirschstein predoctoral National Research Service Award (NRSA) to RY (grant number 1F31MH105161-01A1).

### Conflict of interest statement

The authors declare that the research was conducted in the absence of any commercial or financial relationships that could be construed as a potential conflict of interest.
